# Transcription factor Six2 induces a stem cell‐like phenotype in renal cell carcinoma cells

**DOI:** 10.1002/2211-5463.12721

**Published:** 2019-09-19

**Authors:** Na Cheng, Hongjuan Li, Yan Han, Shuzhen Sun

**Affiliations:** ^1^ Pediatrics Shandong Provincial Qianfoshan Hospital Jinan Shandong China; ^2^ Department of Pediatric Nephrology and Rheumatism and Immunology Shandong Provincial Hospital Affiliated to Shandong University Jinan China

**Keywords:** *nanog*, renal cell carcinoma, Six2, sox2, stemness

## Abstract

Renal cell carcinoma (RCC) accounts for 2–3% of adult malignant tumors, and the incidence of RCC worldwide has increased by about 2% over the past two decades. The homeobox protein Six2 has been shown to promote the stemness of breast cancer cells and play a role in kidney development, but its involvement in RCC progression has not previously been investigated. Here, we found that *six2* expression was significantly increased in RCC tissues and negatively correlated with the overall survival of patients with RCC. In addition, *six2* expression exhibited a remarkably higher level relative to that in normal renal cells. Functional experiments showed that *six2* knockdown attenuated the stemness of RCC cells, which was evident by decreased spheroid formation ability and stemness marker (*sox2* and *nanog*) expression. Mechanistic studies indicated that Six2 directly bound to the enhancer of *sox2*, promoting *sox2* expression and downstream effector expression of *nanog*. Furthermore, overexpression of *sox2* rescued the inhibitory effects of *six2* on the stemness of RCC cells. Notably, *six2* expression is positively correlated with *sox2* and *nanog* expression in RCC tissues. Collectively, our results point toward a *six2/sox2* axis responsible for RCC cell stemness.

**Abbreviations**
CSC: cancer stem cell
kd: knockdown
oe: overexpression
OS: overall survival
qPCR: quantitative real‐time PCR
RCC: renal cell carcinoma
*six2*‐kd: kd lentivirus vector for *six2*

Renal cell carcinoma (RCC) accounts for 2–3% of adult malignant tumors, and the incidence of RCC worldwide has increased by about 2% over the past two decades 1. RCC is heterogeneously prone to local recurrence or distant metastasis after surgical resection 2. Importantly, RCC is not sensitive to radiotherapy and chemotherapy; the traditional treatment of advanced RCC is based on *IL‐2* and *IFN‐α* immunotherapy, but the effective rate for patients is only 5–27%, and the adverse effects are great; in recent years, a series of targeted drugs has been developed and rapidly applied in clinic 3. However, most of the patients display resistance in the late stage; these results emphasize the urgent need to find novel targets for RCC treatments or prognosis.

Cancer stem cells (CSCs) or cells with stemness have been proved to contribute to cell heterogeneity, tumor recurrence, metastasis and drug resistance 4. Many studies have revealed the critical modulators of RCC cell stemness. For example, Wei *et al*. 5 showed that *SIRT2* is highly expressed in RCC cells with stemness, and knockdown (kd) of *SIRT2* reduced the stemness and chemoresistance of RCC cells; Huang *et al*. 6 demonstrated that Galectin‐3 could augment RCC stemness via up‐regulating *CXCR2* expression; and Xiao *et al*. 7 showed that Notch signaling is essential for maintaining the stemness of RCC cells via the *SDF‐1/CXCR4* axis. However, there are still no drugs that could effectively kill stem‐like RCC cells, which means other biomarkers or signaling is critical for RCC stemness. Mutations of the transcription factor *six2* have been associated with renal hypodysplasia, renal cysts or vesicoureteric reflux and provoked to be tested in patients with isolated congenital abnormalities of kidneys 8. Further studies have shown that *six2* promotes the progenitor maintenance of nephron progenitors 9, 10, and *six2* is activated in renal neoplasms and influences cellular proliferation and migration 11. Because the functional traits between progenitors and CSCs are similar, *six2* has been shown to promote the stemness of breast cancer cells 12, 13. However, the roles of *six2* in RCC tumor progression are still unclear.

In this study, we showed that *six2* expression was significantly increased in RCC tissues and negatively correlated with the overall survival (OS) of patients with RCC. Further functional experiments indicated that kd of *six2* attenuated the stemness of RCC cells. Mechanistic studies revealed that Six2 directly bound to the enhancer of *sox2*, a critical regulator for CSC progression, which is responsible for *six2*‐induced stemness of RCC cells. Our results suggest that *six2* may be a potential target for RCC treatments or prognosis.

## Materials and methods

### Clinical samples and cell lines

Twenty‐six pairs of RCC and normal adjacent paraffin‐embedded tissue samples were obtained from the Qianfoshan Hospital of Shandong Province between March 2016 and September 2018. The experiments were undertaken with the understanding and written consent of each subject. Written informed consent from all patients and approval of the hospital ethics review committees were obtained. The study methodologies conformed to the standards set by the Declaration of Helsinki. RCC cell lines SLR23 and SLR20 and human renal epithelial cell line HREpiC were purchased from the Chinese Academy of Sciences Cell Bank. All of the cell lines were cultured in 1640 medium (Thermo Fisher Scientific, Waltham, MA, USA) containing 10% FBS (Thermo Fisher Scientific), 80 U·mL^−1^ penicillin and 0.08 mg·mL^−1^ streptomycin under humidified atmosphere with 5% CO_2_ at 37 °C.

### Data mining

The r2: Genomics Analysis and Visualization Platform (http://r2.amc.nl) was used for analyzing the correlation between *six2* expression and the OS of patients with RCC. In this platform, the only two kidney tumor datasets, Tumor Kidney Renal Clear Cell Carcinoma (The Cancer Genome Atlas; *n* = 533) and Tumor Renal Papillary Cell Carcinoma (The Cancer Genome Atlas; *n* = 290), were used for analysis, and the minimum group size was six (Gene ID: 10736).

### Lentivirus package

The lentivirus package was constructed by Shanghai Genechem Co., Ltd. (Shanghai, China). The kd lentivirus vector for *six2* and overexpression (oe) lentivirus vector for *sox2* were designated *six2*‐kd and *sox2*‐oe, respectively. In addition, control empty lentivirus vector served as a control group in this work. The stable cell lines with *six2*‐kd were screened by culturing with puromycin.

### Quantitative real‐time PCR

Total RNAs from tissues and cells were extracted with TRIeasyTM Total RNA Extraction Reagent TRIeasyTM (YEASEN, Shanghai, China) following the manufacturer's recommendation. Then, cDNA was reversely synthesized using HiScript® Q Select RT SuperMix for quantitative real‐time PCR (qPCR; Vazyme, Nanjing, China) according to the standard procedure. Quantitative RT‐PCR was performed on the StepOne Plus PCR system with AceQ® Universal SYBR® qPCR Master Mix (Vazyme). Glyceraldehyde‐3 phosphate dehydrogenase served as an internal reference. The relative expression levels of transcripts were calculated using the 2^−△△ct^ method.

### Western blot

The detailed procedure was described previously 14. The primary antibodies against these proteins were used: *six2* (1 : 1000; Wanleibio), *sox2* (1 : 1000; Wanleibio, Shenyang, China), *nanog* (1 : 1000; Proteintech, Wuhan, China) and β‐actin (1 : 5000; Beyotime, Beijing, China).

### Spheroid formation assay

The detailed procedure was mentioned in the previous work 15. In brief, RCC cells were cultured in ultra‐low attachment 24‐well plates (Corning, Union City, CA, USA) at 1000 cells per well with Dulbecco's modified Eagle's medium/F12 medium supplemented with 1× B27 (Sigma, St. Louis, MO, USA), 20 ng·mL^−1^ basic fibroblast growth factor (Sigma), 20 ng·mL^−1^ epidermal growth factor (Sigma), 4 μg·mL^−1^ heparin (Sigma) and antibiotics at 37 °C under a 5% humidified CO_2_ atmosphere. After 10 days, the number and size of spheroid were evaluated and quantified under a microscope.

### Luciferase reporter assay

*Sox2* DNA contains two evolutionally conserved enhancers, SRR1 and SRR2. The SRR2 domain was inserted into the pGL3‐Enhancer vector, named pGL3‐*sox2*. The empty vector served as a control vector. In addition, we constructed a single point mutation (T27G) within the putative *six2* binding region of the SRR2 enhancer based on a *six2* binding site that was previously identified via Mut Express MultiS Fast Mutagenesis Kit (Vazyme) 16, named pGL3‐*sox2*‐T27G. The pGL3‐*sox2*, pGL3‐*sox2*‐T27G or empty vector and *Renilla* luciferase construct were cotransfected into RCC cells with *six2* stable oe using Lipofectamine 3000 (Thermo Fisher Scientific) following the manufacturer's recommendation. Seventy‐two hours later, cell lysates were collected, and the luciferase activity was measured using the Dual‐Luciferase Reporter Assay System (Promega, ​Madison, WI, USA). The activity of *Renilla* luciferase was used for normalization.

### ChIP‐qPCR

ChIP‐qPCR analysis was performed using the ChIP kit (Cat No. ab500; Abcam, Cambridge, MA, USA) according to the standard procedure. DNA produced using this kit was used for detecting the enrichment of SRR2 of *sox2* by qPCR assay. The ChIP antibody for *six2* and relative negative antibody were purchased from Proteintech (Cat No. 11562‐1‐AP).

### Cell viability

Cell viability was assessed by the Cell Counting Kit‐8 (Gilson, Middleton, WI, USA) assay. Cells with different treatments were plated in a 96‐well plate at the density of 3000 cells per well and incubated for 24, 48 and 72 h. The number of viable cells was evaluated by the Cell Counting Kit‐8, and 600 nm absorbance was measured using a Microplate Reader (BIO‐TEK, Winooski, VT, USA). All values were normalized to the *A*_600_ value of the control group, which was regarded as 1.

### Statistical analysis

Data analysis was carried out using graphpad prism 6 (Version X; La Jolla, CA, USA). Standard error mean calculations were used for generating error bars. An unpaired one‐tailed Student's *t*‐test was performed to assay the significance between two groups. One‐way ANOVA followed by a Bonferroni comparison was used for experiments with three or more conditions. Asterisks represent the significance of difference from the control group: *P < 0.05, **P < 0.01.

## Results

### *Six2* expression is significantly increased in RCC tissues and cells, and negatively correlated with the OS of patients with RCC

**Figure 1:**
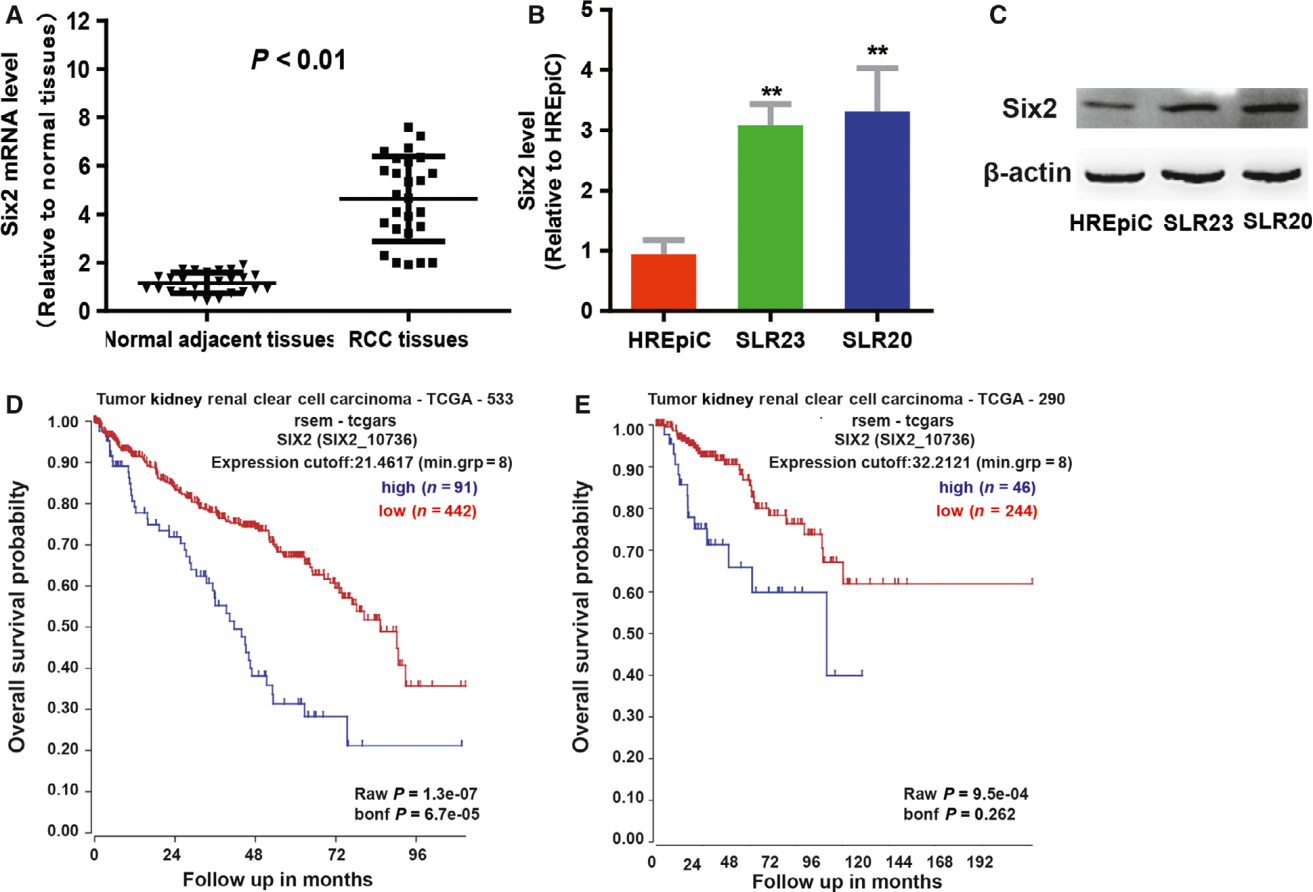
*Six2* expression is significantly increased in RCC tissues and cells, and negatively correlated with the OS of patients with RCC. (A) *Six2* mRNA level was examined in RCC and normal adjacent tissues by qPCR assay. (B) *Six2* mRNA level was detected in RCC and normal renal epithelial cells. (C) *Six2* protein level was measured in RCC and normal renal epithelial cells. (D, E) The correlation between *six2* expression and the OS of patients with RCC was evaluated in two datasets from the r2 platform. The difference was assayed using one‐way ANOVA with the Tukey‐Kramer post‐test. Data were presented as the mean ± SD. **P < 0.01 versus normal adjacent tissues or HREpiC. bonf, Bonferroni; grp, group; min., minimum; TCGA, The Cancer Genome Atlas.

First, we detected *six2* expression in RCC and normal adjacent tissues, and found that *six2* was significantly increased in RCC tissues compared with that in normal adjacent tissues (Fig. 1A). In agreement, *six2* displayed a higher level in RCC cells relative to the normal renal epithelial cells (Fig. 1B,C). Notably, analysis on the two datasets in the r2 platform showed that *six2* expression was negatively correlated with the OS of patients with RCC (Fig. 1D,E). These results indicate that *six2* may hold promoting roles in RCC progression.

### *Six2*‐kd attenuates the stemness of RCC cells

**Figure 2:**
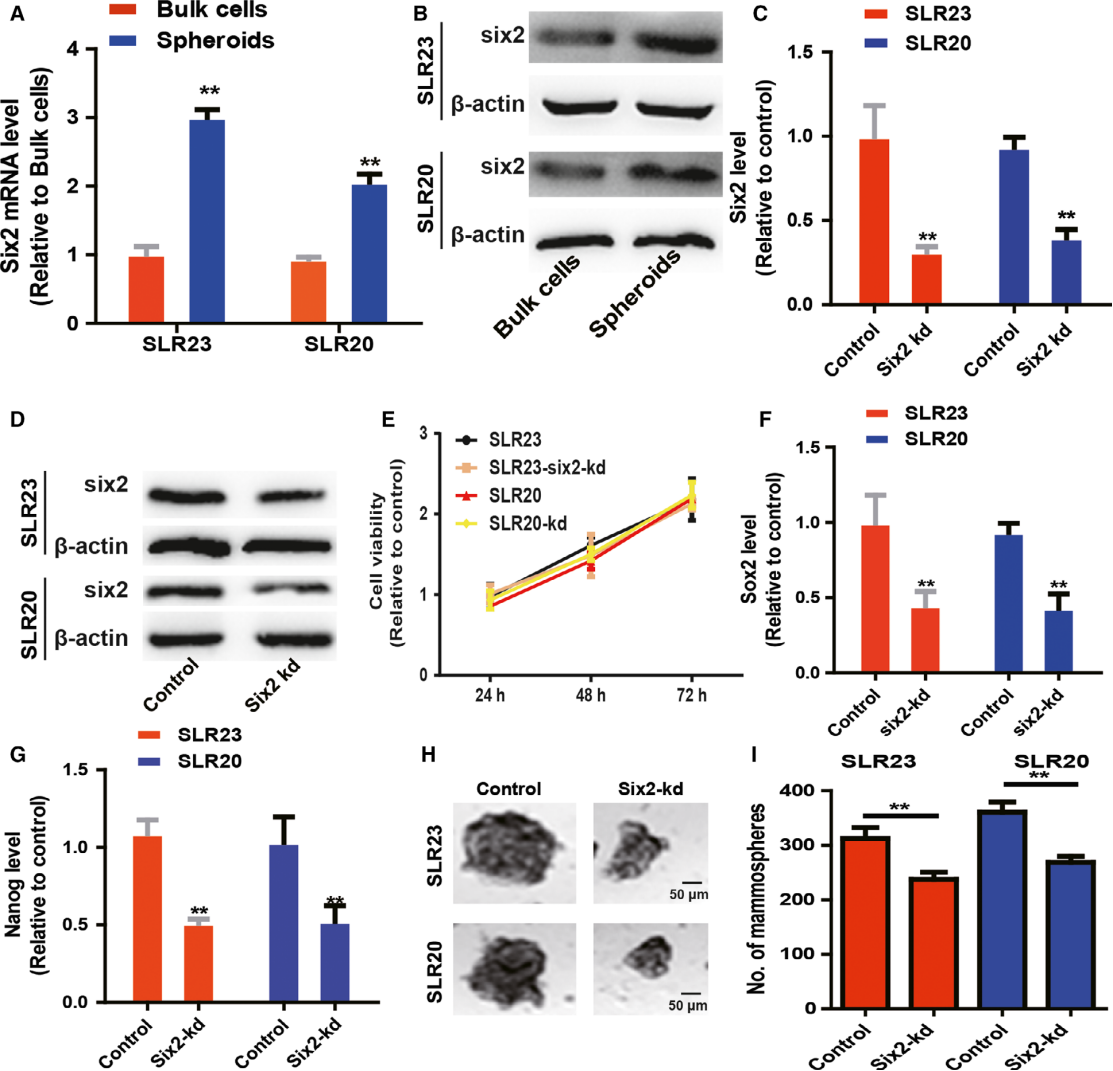
*Six2*‐kd attenuates the stemness of RCC cells. (A, B) *Six2* expression was detected in RCC bulk cells and spheroid cells. (C, D) *Six2* expression was examined in RCC cells with or without *six2*‐kd infection. (E) RCC cells with or without *six2*‐kd were assessed to detect cell viability. (F, G) *Sox2* and *nanog* expression was measured in the cells described in (C). (H, I) The capacity of spheroid formation was evaluated in the cells depicted in (C). The difference was assayed using one‐way ANOVA with the Tukey‐Kramer post‐test. Scale bars, 50 μm. Data were presented as the mean ± SD. **P < 0.01 versus control.

Because CSCs contribute to tumor progression, recurrence and metastasis, we wondered whether *six2* facilitates the stemness of RCC cells. First, we detected the *six2* level in nonadherent RCC spheroid cells and found that *six2* exhibited a remarkably higher level relative to the adherent cells (Fig. 2A,B). Because *six2* expression was higher in RCC cells and tissues, we chose to knock down *six2* expression in RCC cells by lentivirus infection, and stable cell line construction, qPCR and western blot confirmed the kd efficiency of *six2*‐kd (Fig. 2C,D). Notably, *six2*‐kd had no effects on RCC cell viability (Fig. 2E). As expected, *six2*‐kd reduced the expression of stemness regulators (*sox2* and *nanog*; Fig. 2F,G). In addition, spheroid formation ability was attenuated by *six2*‐kd, characterized as the decrease of spheroid size and number (Fig. 2H,I). Therefore, these results demonstrate that *six2* positively regulates the stemness of RCC cells.

### Six2 directly binds to the SRR2 domain of *sox2* and thus promotes its transcriptional activity

**Figure 3:**
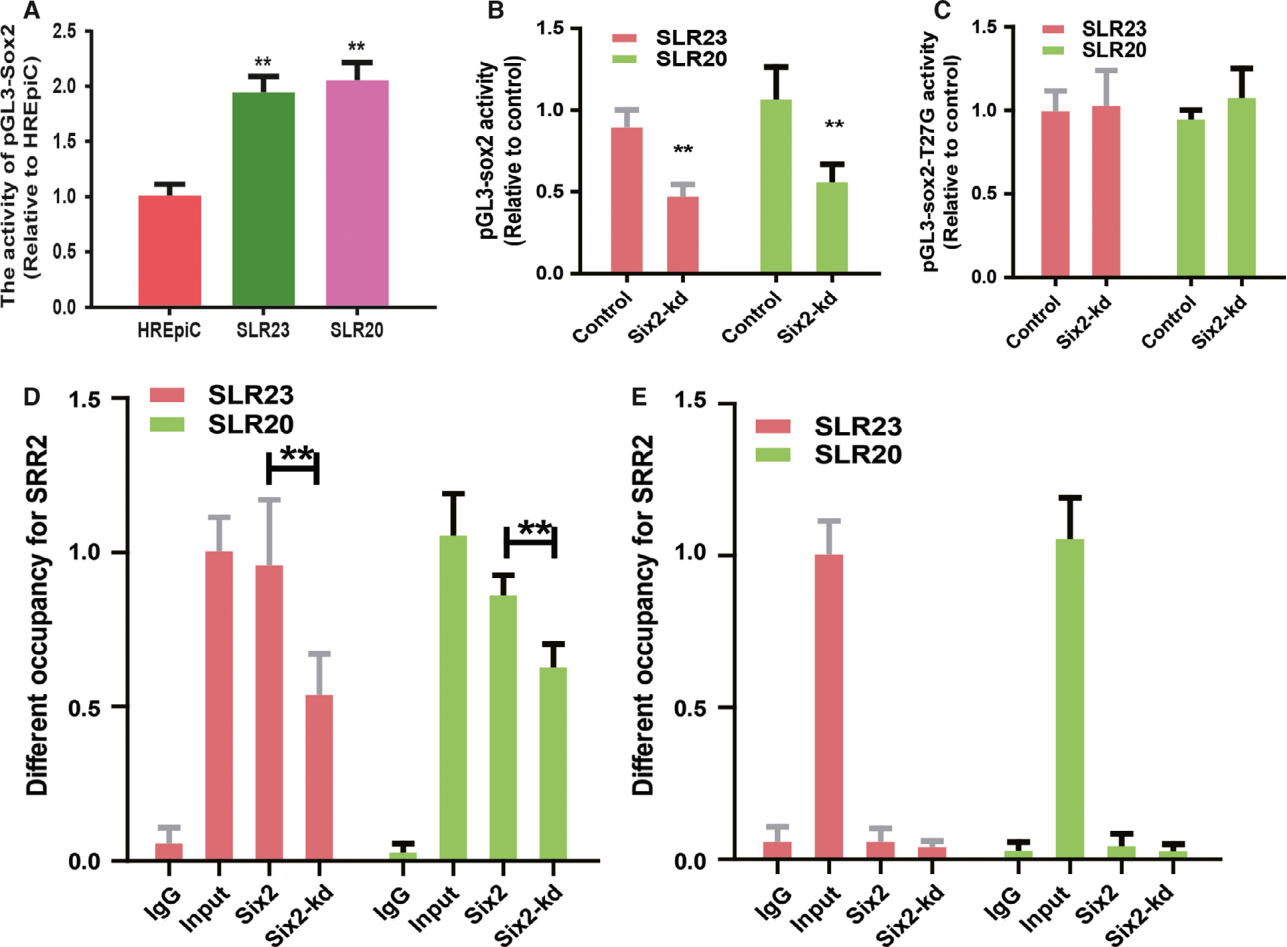
*Six2* directly binds to the SRR2 domain of *sox2* and thus promotes its transcriptional activity. (A) The luciferase activity of pGL3‐*sox2* activity was detected in RCC cells and normal HREpiC cells. (B) The luciferase activity of pGL3‐*sox2* activity was detected in RCC cells with or without *six2*‐kd. (C) The luciferase activity of pGL3‐*sox2*‐T2G was examined in the cells described in (B). (D) The occupancy of SRR2 was determined in the ChIP‐derived DNA as indicated. (E) The occupancy of the region approximately 5 kb downstream of the SRR2 region was measured in the ChIP‐derived DNA as indicated. The difference was assayed using one‐way ANOVA with the Tukey‐Kramer post‐test. Data were presented as the mean ± SD. **P < 0.01 versus control. IgG, immunoglobulin G.

Then, we explored the mechanism by which *six2* regulates the stemness of RCC cells. Two recent studies showed that *six2* promotes the stemness of breast cancer cells via different mechanisms; one indicated that *six2* activates the competing endogenous RNA network between CYP4Z1 and pseudogene CYP4Z2P via directly binding to their promoters 17, and another work demonstrated that Six2 directly binds to the *sox2* SRR2 enhancer region 18. Because CYP4Z1 and pseudogene CYP4Z2P were not expressed in RCC 19, we assumed that Six2 might promote the stemness of RCC cells via directly binding to the *sox2* SRR2 enhancer region. As expected, the basal pGL3‐*Sox2* activity was higher in RCC cells than that in normal HREpiC cells, and *six2*‐kd reduced the luciferase activity of pGL3‐*sox2* in RCC cells, whereas the activity of pGL3‐*sox2*‐T27G was unaffected (Fig. 3A–C). Further, we performed ChIP‐qPCR and found that the SRR2 region was enriched in DNA pulled down by anti‐Six2, whereas *six2*‐kd reduced the SRR2 enrichment (Fig. 3D). To further specify the binding region of SRR2, we performed ChIP‐qPCR to analyze the enrichment of a region approximately 5 kb downstream of the SRR2 region, where no Six2 binding sites exist. As shown in Fig. 3E, the enrichment of Six2 observed in the SRR2 region was lost. Taken together, these results suggest that Six2 indeed directly binds to the SRR2 region of *sox2* in RCC cells.

### *Six2* regulates the stemness of RCC cells dependent on *sox2*

**Figure 4:**
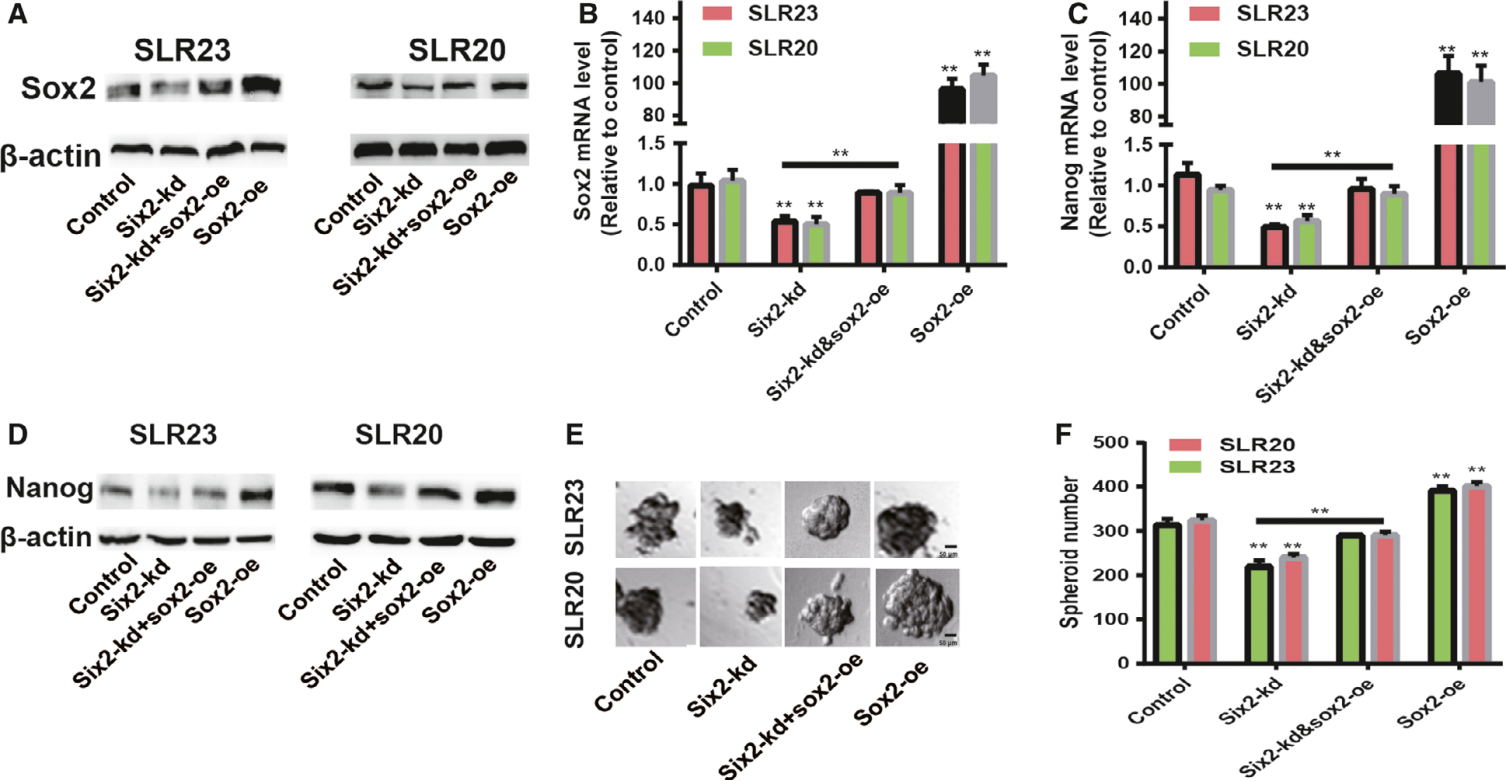
*Six2* regulates the stemness of RCC cells dependent on *sox2*. (A, B) *Sox2* protein and mRNA levels were examined in RCC cells with *six2*‐kd plus *sox2*‐oe or not. (C, D) *Nanog* mRNA and protein levels were measured in the cells described in (A). (E, F) The spheroid formation ability was evaluated in the cells depicted in (A). The difference was assayed using one‐way ANOVA with the Tukey‐Kramer post‐test. Scale bars, 50 μm. Data were presented as the mean ± SD. **P < 0.01 versus control.

We further investigated whether *six2* regulates the stemness of RCC cells through *sox2*. *Sox2* was overexpressed in RCC cells with *six2* stable kd via lentivirus infection, and the infection efficiency was confirmed by western blot and quantitative RT‐PCR (Fig. 4A,B). As expected, the decreased expression of another stemness master regulator *nanog* was rescued by *sox2*‐oe (Fig. 4C,D). In addition, *six2*‐kd‐mediated decrease of spheroid formation ability was partially reversed by *sox2*‐oe (Fig. 4E,F). Collectively, our results demonstrate that *six2* positively regulates the stemness of RCC cells in a *sox2*‐dependent manner.

### *Six2* exhibits a positive correlation with the stemness markers

**Figure 5:**
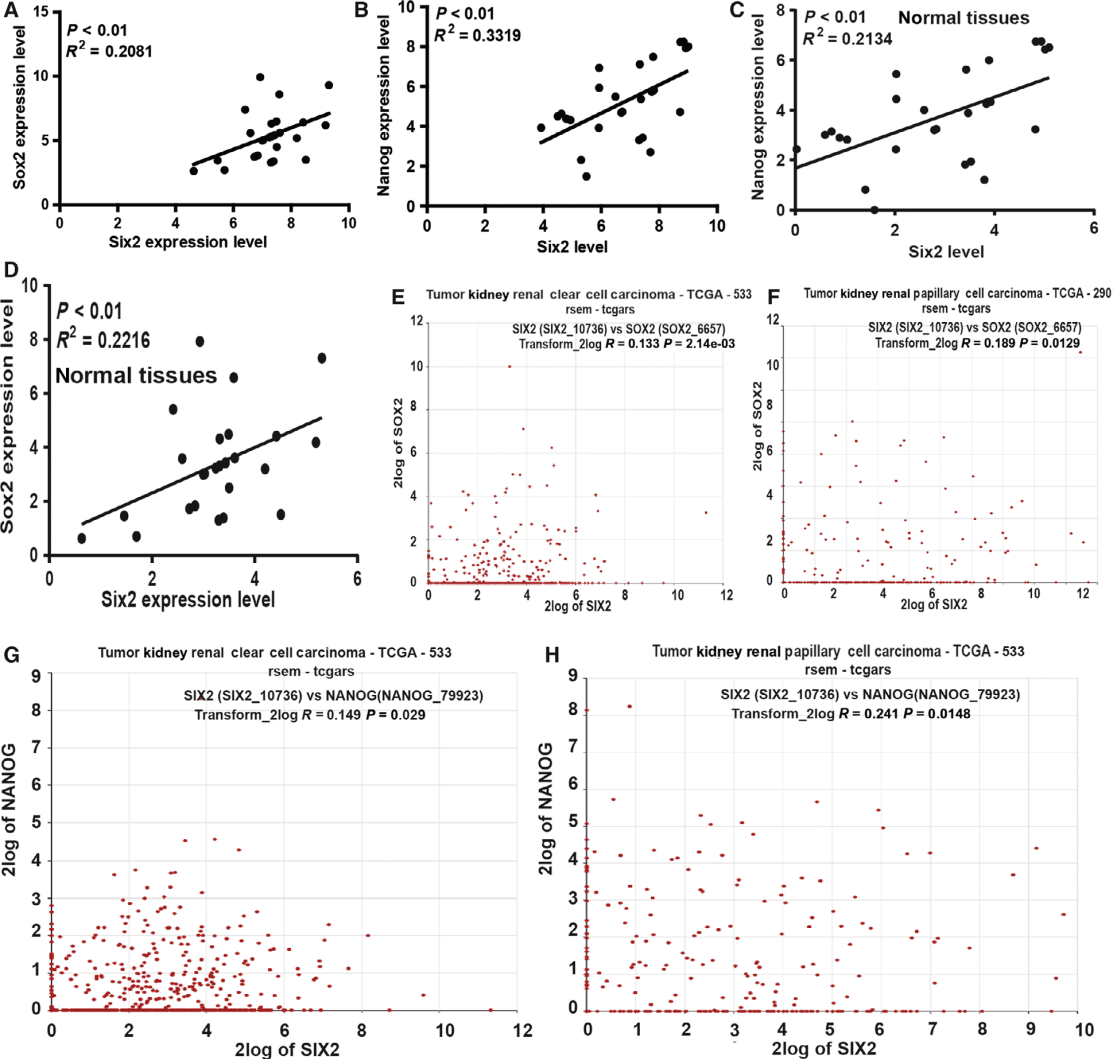
*Six2* exhibits a positive correlation with the stemness markers. (A) The correlation between *six2* and *sox2* expression was evaluated in RCC tissues. (B) The correlation between *six2* and *nanog* expression was examined in RCC tissues. (C, D) The correlation between *six2*,* sox2* and *nanog* expression was determined in normal tissues. (E, F) The correlation between *six2* and *sox2* expression was determined in two RCC datasets from the r2 platform. (G, H) The correlation between *six2* and *nanog* expression was analyzed in two RCC datasets from the r2 platform. TCGA, The Cancer Genome Atlas.

Finally, to further confirm our conclusion, we detected the correlation between *six2*,* sox2* and *nanog* expression in clinical samples and online datasets from the r2 platform. We found that the expression of *six2* and *sox2* or *nanog* exhibited a positive correlation in clinical RCC and normal tissues (Fig. 5A–D). Notably, a consistent result was observed in the online dataset from the r2 platform (Fig. 5E–H).

## Discussion

This work indicated that transcription factor Six2 positively regulated the stemness of RCC cells via directly binding to the SRR2 enhancer of stemness master regulator *sox2*. Although the promoting roles of *six2* have been established in other tumors, to our knowledge, this is the first work to reveal the roles of *six2* in RCC cell stemness.

CSCs have been regarded as the root of tumor progression, recurrence, metastasis and drug resistance. A previous study showed that salinomycin exhibits a specific killing capacity on CSCs by sequestering iron in lysosomes 20. In addition, salinomycin could suppress the stemness and induce the apoptosis on human ovarian CSCs 21. Notably, salinomycin reduces the stemness and epithelial‐mesenchymal transition process of RCC cells 22. Moreover, Zheng *et al*. 23 showed that a CD13‐targeting peptide integrated protein inhibits human liver cancer growth by killing CSCs. These results suggest that there are drugs that specifically kill CSCs; this should be further explored in the future. In the present study, we found that transcriptional factor *six2* positively regulates the stemness of RCC cells. Evaluating the previous works that reveal that *six2* promotes the stemness of breast cancer cells 17, 18, we wonder whether the promoting roles of *six2* on cancer cell stemness are common in other tumors. Future work should be performed to resolve this issue, which could facilitate *six2* as the drug target.

Notably, the previous study has shown that *nanog* acts as the downstream effector of *sox2* in breast cancer 24; this result is consistent with our results in this work. In addition, the roles of *six2* in nephrogenesis, coupled with *six2* expression in RCC, indicate that *six2* may be a critical regulator of both renal cell stemness and RCC stemness, and it will be of interest to determine whether *six2* similarly regulates *sox2* and the downstream effector *nanog* in the context of normal renal development. Importantly, we must admit that *six2*, like *sox2*, is a transcriptional factor and thus not an ideal drug target. Collectively, this work identifies that *six2* plays a critical role in promoting RCC cell stemness through *six2*/*sox2* signaling. Notably, the relationship between *six2* and *sox2* or *nanog* expression is positively relevant, and *six2* expression is negatively correlated with the OS of patients with RCC. Therefore, it will be important to identify more targetable downstream effectors of *six2* that are critical in mediating its ability to promote RCC cell stemness.

## Conflict of interest

The authors declare no conflict of interest.

## Author contributions

NC, HL, YH and SS conceived and designed the project. NC, HL and YH acquired the data. NC and HL analyzed and interpreted the data. NC and HL wrote the paper
